# Efficacy and Safety of Pentoxifylline in Major Depressive Disorder: A Systematic Review and Meta-Analysis of Randomized Controlled Trials

**DOI:** 10.1192/bjo.2025.10134

**Published:** 2025-06-20

**Authors:** Omar Kassar, Osama Hassan, NourAllah Farag, Abdullah Selim, Moaz Elsayed Abouelmagd

**Affiliations:** ^1^Faculty of Medicine, Alexandria University, Alexandria, Egypt; ^2^Central and North West London NHS Foundation Trust, London, United Kingdom; ^3^Faculty of Medicine, Zagazig University, Zagazig, Egypt; ^4^Faculty of Medicine, Suez University, Suez, Egypt; ^5^Faculty of Medicine, Cairo University, Cairo, Egypt

## Abstract

**Aims:** Major depressive disorder (MDD) may be linked to broader pathophysiological pathways such as oxidative stress, inflammation, vascular dysfunction, and neuroplasticity alterations. Pentoxifylline (PTX), a pleiotropic drug, targets all these pathways through non-specific phosphodiesterase (PDE) inhibition. This is the first systematic review and meta-analysis to examine the role of PTX in major depressive disorder.

**Methods:** A comprehensive search of electronic databases, including PubMed, Scopus, Cochrane, and Web of Science, was performed in October 2024. We included only randomized controlled trials (RCTs), and their data were extracted and analysed using Reman 5.4 software. The inclusion criteria as follows: adult patients diagnosed with MDD were included as the population. The intervention considered was pentoxifylline, either alone or in combination with selective serotonin reuptake inhibitors. Comparators included placebo, either alone or combined with SSRIs. Eligible studies needed to report outcomes such as the Hamilton Depression Rating Scale (HAM-D).

**Results:** Four RCTs with 318 patients were included in the study. PTX showed a statistically significant improvement in HAM-D scores at the primary endpoint compared with the placebo (MD=−3.84, 95% CI [−4.87 to −2.81], p<0.00001). Moreover, PTX showed a statistically significant increase in serotonin and BDNF levels (MD=20.76 ng/mL, 95% CI [5.49 to 36.04], p=0.008; and MD=10.83 ng/mL, 95% CI [−0.22 to 21.88], p=0.05, respectively) and a statistically significant decrease in TNF-α and IL-6 levels (MD=−3.24 pg./mL, 95% CI [−4.12 to −2.36], p<0.00001; and MD=−2.64 pig/mL, 95% CI [−3.79 to −1.48], p<0.00001, respectively). There was no statistically significant difference between the PTX and placebo in any of the reported side effects including nausea, vomiting, headache, diarrhoea, increased appetite, and sexual dysfunction.

**Conclusion:** The study findings suggest that PTX may be effective and safe as an adjuvant antidepressant agent in patients with MDD, demonstrating a significant reduction in HAM-D scores. The results of this study need to be interpreted with caution considering several limitations.

